# Keratocyte Apoptosis and Not Myofibroblast Differentiation Mark the Graft/Host Interface at Early Time-Points Post-DSAEK in a Cat Model

**DOI:** 10.1371/journal.pone.0075623

**Published:** 2013-09-30

**Authors:** Adam J. Weis, Krystel R. Huxlin, Christine L. Callan, Margaret A. DeMagistris, Holly B. Hindman

**Affiliations:** 1 University of Rochester School of Medicine and Dentistry, Rochester, New York, United States of America; 2 Flaum Eye Institute, University of Rochester Medical Center, Rochester, New York, United States of America; 3 Center for Visual Science, University of Rochester, Rochester, New York, United States of America; Bascom Palmer Eye Institute, University of Miami School of Medicine, United States of America

## Abstract

**Purpose:**

To evaluate myofibroblast differentiation as an etiology of haze at the graft-host interface in a cat model of Descemet’s Stripping Automated Endothelial Keratoplasty (DSAEK).

**Methods:**

DSAEK was performed on 10 eyes of 5 adult domestic short-hair cats. *In vivo* corneal imaging with slit lamp, confocal, and optical coherence tomography (OCT) were performed twice weekly. Cats were sacrificed and corneas harvested 4 hours, and 2, 4, 6, and 9 days post-DSAEK. Corneal sections were stained with the TUNEL method and immunohistochemistry was performed for α-smooth muscle actin (α-SMA) and fibronectin with DAPI counterstain.

**Results:**

At all *in vivo* imaging time-points, corneal OCT revealed an increase in backscatter of light and confocal imaging revealed an acellular zone at the graft-host interface. At all post-mortem time-points, immunohistochemistry revealed a complete absence of α-SMA staining at the graft-host interface. At 4 hours, extracellular fibronectin staining was identified along the graft-host interface and both fibronectin and TUNEL assay were positive within adjacent cells extending into the host stroma. By day 2, fibronectin and TUNEL staining diminished and a distinct acellular zone was present in the region of previously TUNEL-positive cells.

**Conclusions:**

OCT imaging consistently showed increased reflectivity at the graft-host interface in cat corneas in the days post-DSAEK. This was not associated with myofibroblast differentiation at the graft-host interface, but rather with apoptosis and the development of a subsequent acellular zone. The roles of extracellular matrix changes and keratocyte cell death and repopulation should be investigated further as potential contributors to the interface optical changes.

## Introduction

Despite its many advantages over full thickness penetrating keratoplasty (PK) [1], [2], endothelial keratoplasty can yield sub-optimal post-operative visual acuity. [3] Scatterometry and adaptive optics analyses suggest that corneal haze is a likely contributor to this sub-optimal vision. [1], [4] *In vivo* human studies have investigated the locations and roles of corneal haze in visual acuity and other measures of visual function. [5], [6] The graft-host interface has been identified as an area of increased corneal haze post-DSAEK. [7] Interface reflectance, as measured by *in vivo* confocal imaging, decreases with time post-DSAEK and has been correlated with improvements in post-operative visual acuity. [8] However, while interface haze appears to be implicated in visual acuity limitations post-DSAEK, the cause of this interface haze remains unknown.

In the normal cornea, keratocytes are quiescent cells that contribute to corneal clarity by maintaining the structure of the collagen lamellae and the composition of the extracellular matrix. In response to wounding stimuli, keratocytes become activated and differentiate into a myofibroblast phenotype. [9], [10] Myofibroblasts require several proteins necessary for successful pro-fibrotic wound closure and healing including alpha smooth muscle actin (α-SMA) [11], [12] and extracellular fibronectin. [13].

Myofibroblasts are less transparent than non-activated keratocytes,[14]–[16] and they further decrease stromal transparency by altering the organization and composition of the extracellular matrix.[17]–[19] In models of wound healing such as incisional wounds and photoablation, haze has been largely attributed to myofibroblast activity. [15] In this study, we utilized a cat model of DSAEK to test the hypothesis that corneal haze at the post-DSAEK graft-host interface is due to myofibroblasts differentiation and activity.

## Methods

### Ethics Statement

This study adhered to the ARVO Statement for the Use of Animals in Ophthalmic and Vision Research, the guidelines of the University of Rochester Committee on Animal Research (UCAR), and the NIH Guide for the Care and Use of Laboratory Animals. All research was conducted with the approval of our Institutional Animal Care and Use Committee (IACUC), the University of Rochester Committee on Animal Research (UCAR). Our protocol was adhered to all national and international guidelines regarding laboratory animal care and welfare. Pain medications were provided for all surgical procedures, and the cats were closely monitored for signs of distress or discomfort throughout their time on study. Every effort was taken to ensure their good health and minimize distress.

### Surgical Procedure

#### Animals

DSAEK was performed on 10 eyes of 5 normal, adult, domestic, short-hair cats (*Felis cattus*) purchased from Liberty Research, Inc., Waverly, NY.

#### Donor tissue preparation

Fresh enucleated cat globes were placed in 2.5% Chondroitin Sulfate-Dextran (Optisol-GS Corneal Storage Media, Bausch & Lomb; Rochester NY) and shipped overnight on ice from Liberty Research, Inc., Waverly, NY. Donor allograft tissue was prepared the following morning (day of surgery). The corneas were excised from the globes then cut in a lamellar fashion utilizing the Moria microkeratome (Moria Inc.; Doylestown, PA) (300 mm head) and artificial anterior chamber. The posterior lamellae were then punched with a 10.0 mm diameter trephine to account for the larger cat corneal diameter.

#### Surgery

Cats received perioperative oral prednisone with a tapering dosage schedule from 30 mg a day starting 3 days prior to the procedure, to 20 mg at day 8 post-operatively. Anesthesia was induced with an intravenous injection of ketamine HCl (10 mg/kg) and diazepam (0.5 mg/kg), and maintained via isoflurane inhalant (1.5–2.5%) after intubation. Atropine sulfate (0.04 mg/kg) was administered intramuscularly as a pre-anesthetic adjuvant to inhibit respiratory secretions and prevent bradycardia. For pain management and prevention, flunixin meglumine (0.25 mg/kg) was provided intravenously prior to the procedure and by subcutaneous injections 24 hours post-procedure. A pediatric speculum was inserted to retract the nictitating membrane and lid. A superonasal paracentesis was made through which an anterior chamber maintainer was inserted and 10 U/mL preservative-free heparin (Heparin Sodium Injection, USP (10,000 U/mL), Hospira, Inc.;Lake Forest, IL) in Balanced Salt Solution Plus (Alcon Laboratories, Inc.; Fort Worth, TX) was infused. Additional intracameral injections of heparin in balanced salt solution (1000 U/mL) were administered periodically to prevent intraoperative fibrin formation. A 9.5 mm diameter Descemetorhexis was performed with a reverse Sinskey hook and an adapted micro-utrata through a superotemporal paracentesis. Peripheral posterior stromal roughening was performed with a scraper. This supero-temporal corneal paracentesis was then enlarged to 5.5 mm, and the donor tissue inserted over a healon-coated lens glide. Three 8.0 monofilament nylon sutures were used to close the larger supero-temporal corneal incision and 1 suture was used to close the supero-nasal paracentesis. The tissue was centered through external manipulation, and an air bubble was inserted into the anterior chamber to completely inflate the anterior chamber and support graft/host tissue apposition. At the conclusion of each procedure, each eye received a subconjunctival injection of 0.5 cc triamcinolone acetonide (40 mg/ml) and a topical application of pre-mixed cyclopentolate, phenylephrine, and mydriacyl drops. Moxifloxacin and cyclosporine were applied twice daily and prednisolone acetate 1% was applied four times daily. All surgical procedures were performed at the University of Rochester by one surgeon (HBH).

### Optical Imaging

Cats underwent a series of non-invasive, *in vivo,* optical measurements pre-operatively and twice weekly after DSAEK until they were sacrificed at time points between 4 hours and 9 days post-operatively. Cats were anesthetized for imaging with an intramuscular injection of ketamine HCl (5 mg/kg) and dexmedetomadine HCl (0.04 mg/kg). On completion of imaging studies, atipamezole HCl (0.3 mg/kg) was administered by intravenous injection as a reversal agent.

#### Slit lamp examination and photography

Slit lamp examination was performed to assess for graft adherence, corneal clarity, and anterior chamber inflammation.

#### Corneal Optical Coherence Tomography (OCT)

A custom-built, 1310 nm anterior segment optical coherence tomographer (OCT) was used to quantify corneal backscatter light intensity from defined regions and the thicknesses of specified layers within the corneas. A video stream of the central cornea at a rate of eight frames per second was captured, and fifteen images were extracted from each video for analysis.

#### Corneal light backscatter measurements

**Figure 1:**
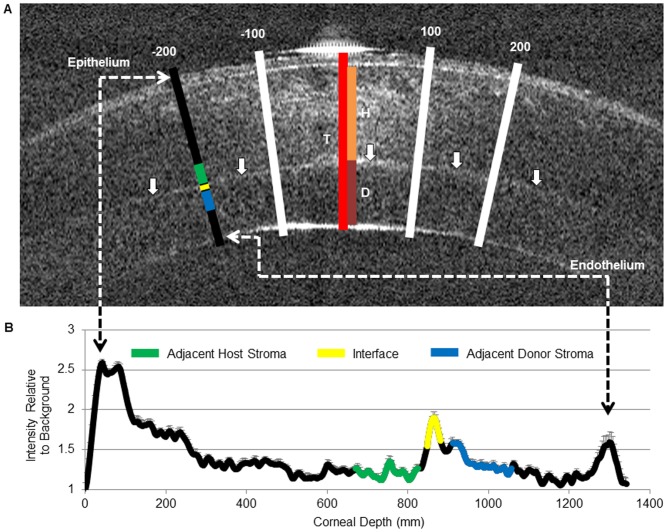
Methods of Corneal Optical Coherence Tomography (OCT) Analysis. (**A**) OCT image of an *in vivo* cat cornea 4 hours after Descemet’s Stripping Automated Endothelial Keratoplasty (DSAEK). Note the greater intensity of backscatter at the graft-host interface (solid arrows) relative to the adjacent stromata. The perpendicular lines superimposed over the OCT image indicate the location of the areas analyzed for backscatter intensity (white and black) and thickness (red, orange, and maroon). Four perpendicular lines (white and black), 20 pixels wide and located +/−100 and +/−200 pixels from the central specular reflection were used to generate a pixel brightness profile. As depicted along the black line, intensity line graphs generated from each of the four perpendicular lines were then used to identify and measure intensity over three defined corneal regions; a 10 pixel thick region of interface (yellow), a 40 pixel thick region of the adjacent host stroma (green), and a 40 pixel thick region of the adjacent donor stroma (blue). Total corneal (red), host stromal (orange), and donor stromal (maroon) thickness measurements were taken in the central cornea as depicted by the central line. (**B**) A representative plot of normalized backscattered light intensity through a cat cornea post-DSAEK at a single time-point. This profile was generated as described above. These graphs were constructed for each of the line locations. Note the intensity peak at the interface (yellow) in comparison to that of the adjacent host (green) and donor stroma (blue).

Corneal haze was measured using ImageJ software to quantify pixel intensity along four, 20-pixel wide (75 µm), lines drawn perpendicular to the corneal surface at −200, −100, 100 and 200 pixels (approximately +/−375 and +/−750 µm) from the center of the anterior cornea (Figure 1A). The area of saturated reflectivity associated with the specular reflection was thereby avoided while still obtaining data as close to the central cornea as possible. Lines were extended perpendicularly from the anterior surface to the posterior surface of the cornea (including graft if present) in the cross-sectional image. Pixel brightness values at each point along the lines were averaged to create a mean intensity profile, the values of which were subsequently normalized to the background intensity. The corresponding data from 15 images obtained from each eye were averaged and plotted in an average pixel intensity line graph for each eye at each time-point (Figure 1B). Intensity line graphs generated from each of the four perpendicular lines were then used to identify and measure intensity over three defined corneal regions: a 10 pixel (37.5 µm) thick region of interface, a 40 pixel (150 µm) thick region of the adjacent host stroma, and a 40 pixel (150 µm) thick region of the adjacent donor stroma. These regions were identified, by correlating epithelial, stromal, interface, and endothelial peaks along the intensity line graphs with corresponding thickness measures taken at the same location. Mean values within interface and adjacent donor and host stromal regions from each of the 4 line graphs were averaged to get a mean intensity for each region (host stroma, interface, and donor stroma) within each eye. The values from each region were then averaged with corresponding data from all eyes at the same time-point to determine average backscatter values for each region at each time-point.

#### Corneal thickness measurements

Image J software was used to measure central corneal thicknesses. Measurements were taken as close to the central pixel as possible while remaining outside the specular reflection (where layer distinctions become blurred). Selected regions were within a distance of +/−94 µm (25 pixels) of the central pixel. Measurements were taken for the total corneal thickness (from the anterior epithelial surface to the posterior edge of the endothelial cell layer), the host stroma (from anterior host stromal surface to the host-donor interface), and the donor stroma (from the host-donor interface to posterior donor surface) (Figure 1A). Corresponding measurements were averaged across the 15 images obtained of each eye at each time-point. The mean total corneal, host stromal, and donor stromal thicknesses for all eyes measured at each time-point were then calculated.

### *In Vivo* Confocal Imaging of Cat Corneas

A Heidelberg Retinal Tomograph (HRTII) confocal microscope with Rostock Cornea Module (Heidelberg Engineering, Inc., Dossenheim, Germany) was used to detect the presence and morphology of keratocytes and to assess for qualitative changes in their density. Additionally, reflectivity of the stroma and morphology of the interface were assessed. Videographic data were recorded with automatic exposure throughout the central thickness of each cornea. Representative images were extracted from individual frames within the interface and from the adjacent 100 µm of stroma on the donor and host side.

### Statistical Analyses

Statistical analyses were performed using Instat Graphpad software (GraphPad InStat version 3.10 for Windows, GraphPad Software, San Diego California USA), which automatically corrects for multiple comparisons. The Kruskal-Wallis test with Dunn’s multiple comparisons tests were used to compare corresponding thickness measures and regional intensity measurements across different time-points. The Wilcoxon Matched-Pairs Signed-Ranks Test was used to compare intensity values between the interface and the adjacent host stroma and donor stroma within each individual time-point. A P value of less than 0.05 was considered statistically significant.

### Post-Mortem Histological Analyses of Cat Corneas

Cats were sacrificed using sodium pentobarbital 7.8% isopropyl alcohol euthanasia solution (Sleepaway, Fort Dodge Animal Health, Fort Dodge, Iowa) at 4 hours and 2, 4, 6 and 9 days post-DSAEK, and the corneas were immediately harvested for histology. Corneas were removed with a limbal edge, drop fixed in 1% paraformaldehyde in 0.1 M PDS (pH 7.4) for 10 minutes, and then transferred to 30% sucrose in 0.1 M PBS for cryoprotection at 4°C for 2 days. The tissue was embedded in OCT (Tissue Tek, Sakura Finetek, Zoeterwoude, The Netherlands) and frozen using liquid nitrogen. The tissue was cut into serial 20 µm sections using a cryostat (2800 Frigocut E, Leica, Nussloch, Germany) at −20°C, mounted on glass slides, and stored at −20°C. Two unoperated control corneas were harvested in a similar fashion from enucleated cat globes and used as experimental negative controls. Sections containing incisional wounds and limbal tissue were used as positive and negative antibody controls.

#### Immunohistochemistry

Corneal sections were fluorescently labeled for α-SMA and fibronectin. Sections were thawed overnight, washed in 0.1 M phosphate buffer, and encircled with a hydrophobic barrier. They were then incubated overnight at 4°C with mouse anti- α-SMA (0.1 µg/mL; Clone 1A4, Thermo Fischer Scientific, Fremont, CA), and rabbit anti-fibronectin (5.0 µg/mL; Millipore Corporation, Temecula, CA) primary antibodies diluted with 1% Triton X-100 in 0.1 M PBS (Sigma Aldrich, St. Louis, Missouri). After washing, sections were incubated for 4 hours at room temperature with goat anti-mouse Alexa-Fluor 555 (4.0 µg/mL; Invitrogen, Carlsbad, California) and goat anti-rabbit Alexa-Fluor 488 (2.0 µg/mL; Invitrogen, Carlsbad, California) secondary antibodies. Sections were then washed and mounted under glass coverslips using mounting media containing 4′6′-diamidino-2-phenylondole dihydrochloride (DAPI) (Vectashield Mounting Medium with DAPI, Vector Laboratories, Burlingame, California). DAPI binds to double-stranded DNA, thus staining intact nuclei and serving as a counterstain.

#### TUNEL assay

A TUNEL assay was used to detect fragmented DNA and identify apoptotic cells. After tissue preparation as above, the staining was performed as instructed using a commercially available kit (ApopTag Red *In Situ* Apoptosis Detection Kit, Chemicon International, Temecula, CA).

#### Image Analysis

Tissue sections were viewed with a Zeiss Axio Imager M1 fluorescence microscope (Zeiss, Gottingen, Germany) and photomicrographs were obtained using an integrated video camera interfaced with a computer running Axiovision Software (AxioVs40 V 4.8.2.0, Carl Zeiss Microimaging GmbH, Oberkochen, Germany). Thick sections were brought into focus by capturing z-stack images and post-processing with the ImageJ Software z project tool.

## Results

### DSAEK in Cats – surgical Outcomes

**Figure 2:**
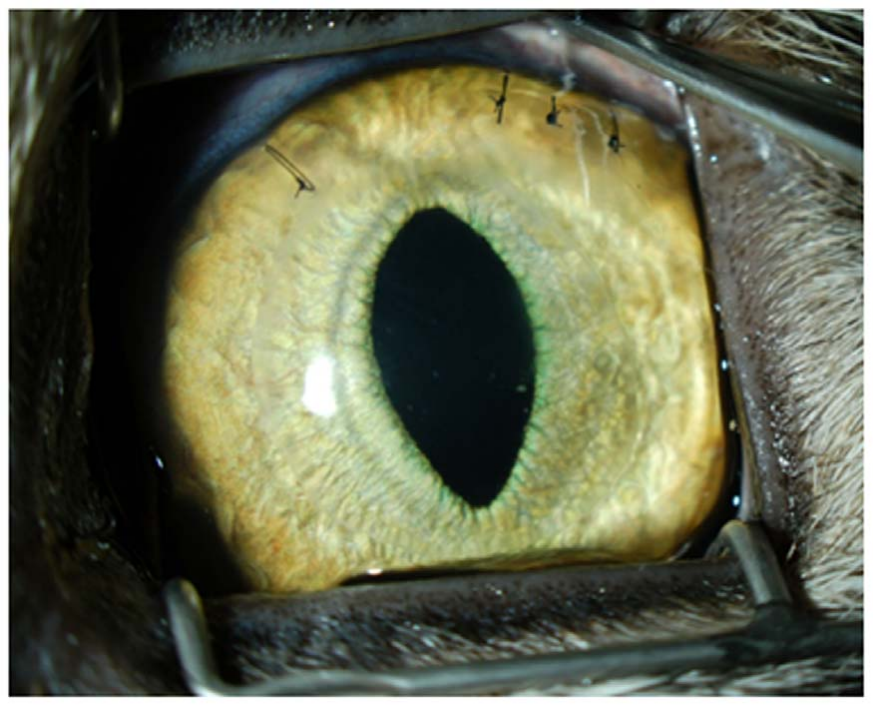
Descemet’s Stripping Automated Endothelial Keratoplasty (DSAEK) Allograft In A Cat. External photograph of the left eye of a cat 6 days post-DSAEK. Note the clear cornea and good iris detail. The anterior chamber air bubble has resolved. Sutures were used to close the paracentesis and corneal incision sites.

**Figure 3:**
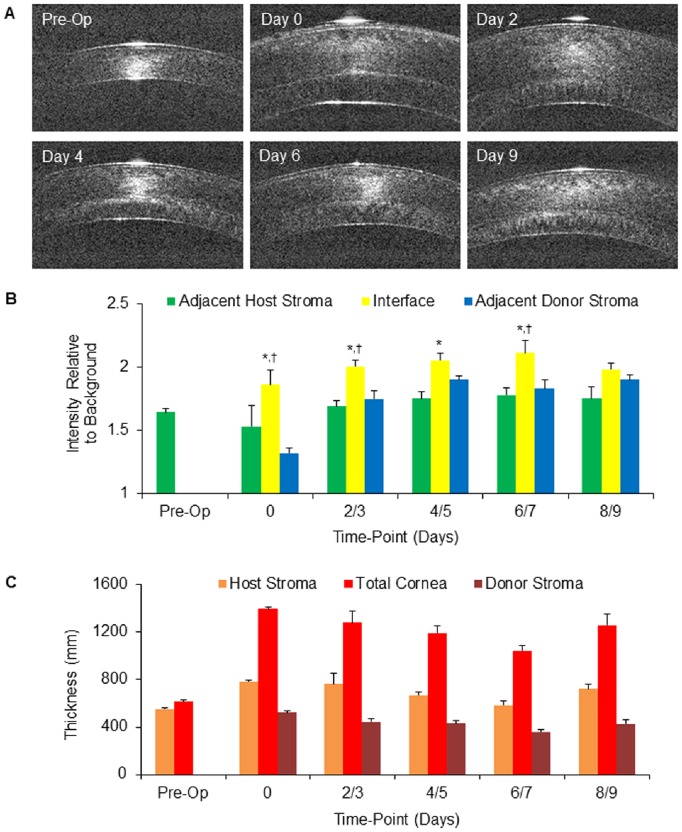
Interface Intensity and Corneal Thickness Post-DSAEK. (**A**) Corneal optical coherence tomography (OCT) images of an *in vivo* cat cornea pre-operatively and at early post-Descemet’s Stripping Automated Endothelial Keratoplasty (DSAEK) time-points. Note the easily discernible post-operative interface between the host and donor stromata appears brighter than the adjacent tissue. The host stroma also has increased thickness immediately post-operatively, which improves with time. (**B**) Corneal OCT image-derived backscatter intensity at the graft-host interface (yellow) in comparison to the adjacent host stroma (green) and donor stroma (blue) at early post-DSAEK time points. Note that the graft-host interface was consistently brighter than the adjacent host stroma and adjacent donor stroma. This result was statistically significant at the majority of time-points; between interface and adjacent host stroma at days 0 (p = 0.0078), 2/3 (p = 0.0053), 4/5 (p = 0.0019) and 6/7 (p = 0.0003), and between interface and adjacent donor stroma at days 0 (p = 0.0078), 2/3 (p = 0.0005), and 6/7 (p = 0.0155). This difference was no longer observed at the 8/9 day time point. * = significant difference between mean interface and mean host stromal intensity. † = significant difference between mean interface and mean donor stromal intensity. (**C**) OCT image-derived corneal thickness measurements across early post-DSAEK time-points. Note the brisk increase in total thickness from pre-operative levels (Pre-Op) to immediate post-operative (0) levels associated with the addition of the donor tissue, and the subsequent gradual decline in total thickness to day 6/7.

All surgeries were performed successfully resulting in clear corneas (Figure 2) with no primary graft failures or tissue dislocations. One cat required an additional suture to be placed at the main wound on post-operative day 2 for a small wound leak. The surgical procedures and follow-up care were otherwise identical between eyes and cats. Slit lamp examinations and corneal OCTs revealed that the host stroma swelled immediately after the surgery (from preoperatively to first postoperative measurement) as demonstrated by an increase in the host stromal thickness (Figure 3A and C). The host stromata and donor stromata subsequently decreased in thicknesses over the following week after the donor tissues adhered demonstrating successful endothelial replacement and corneal deturgescence (Figure 3A and C). After 9 days, increasing anterior chamber inflammation, cellular deposition along the donor endothelium, and corneal thicknesses likely indicated immunologic rejection that ultimately limited our ability to study the transplanted cat corneas beyond the 9-day time-frame reported here.

### Interface Reflectivity Exceeded that of the Adjacent Host and Donor Stroma

The OCT images revealed a graft-host interface that was consistently brighter than the adjacent host stroma and adjacent donor stroma at all time-points examined up to 9 days post-DSAEK (Figure 3A). This result was statistically significant at the majority of time-points; between interface and adjacent host stroma at day 0 (p = 0.0078), day 2/3 (p = 0.0053), day 4/5 (p = 0.0019) and day 6/7 (p = 0.0003), and between interface and adjacent donor stroma at days 0 (p = 0.0078), 2/3 (p = 0.0006), and 6/7 (p = 0.02). The differences were no longer significant at the 8/9 day time point (Figure 3B), possibly due to the small sample size at this time-point.

### Corneal Thicknesses were Stable Across Post-operative Time Points

The host stromal thickness measurements did vary significantly across all time points (p = .01, KW 14.995); however, none of the post-test comparisons were significant. Across the post-operative time points alone, none of the tissue thicknesses (host stromal, donor stromal, or total corneal) varied significantly, although trends of decreasing tissue thicknesses from immediately post-operatively through day 7 were observed (Figure 3C).

### *In vivo* Confocal Imaging Demonstrates an Acellular Zone at the Donor/Host Interface

**Figure 4:**
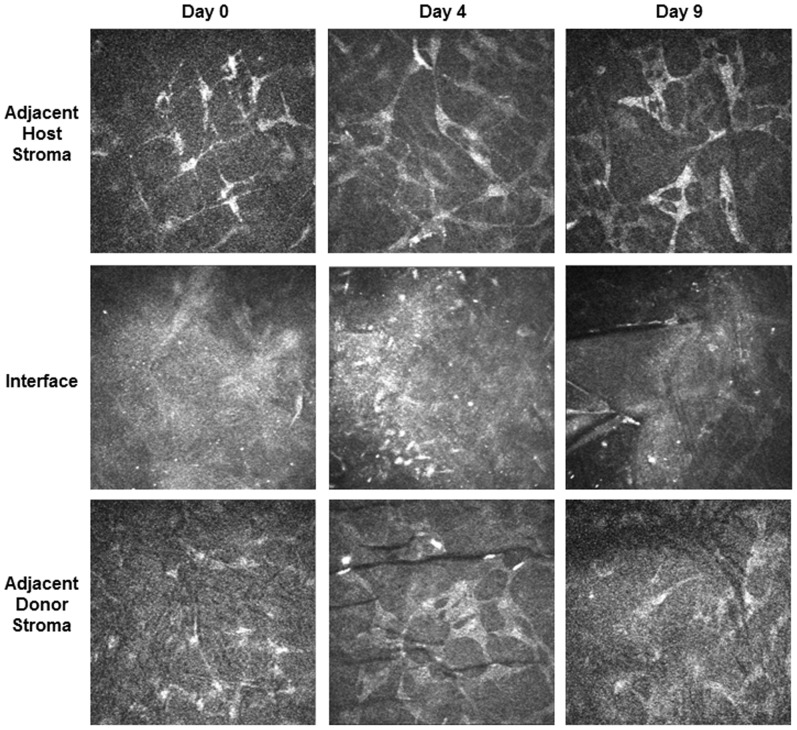
*In vivo* confocal imaging post-Descemet’s Stripping Automated Endothelial Keratoplasty (DSAEK). Confocal images of an *in vivo* cat cornea at day 0 (4/5 hours), day 4, and day 9 post-DSAEK. Note the absence of cells but presence of reflective particles at the graft-host interface. Adjacent host and donor stromal images were taken within 100 µm of the acellular interface. Day 0 adjacent host stromal cells appear to have a blotchy reflectance. The other adjacent stromal cells appear to be a regular network of interconnected broad dendritic keratocytes with less contrast between the cell and clear stroma.

All time-points revealed at least one plane of acellularity at the interface. On day zero, there was a single plane of acellularity at the interface that exhibited both an extensive diffuse reflectivity and the presence of small reflective particles dispersed throughout. Adjacent cells with keratocyte morphology appear to have a blotchy reflective quality. Day 4 revealed a broader acellular zone with a less homogeneous, lacey reflective pattern and the reflective particles at the interface appeared larger, more distinct, and more numerous than on day 0. Cells adjacent to the acellular zone appear as regular, interconnected, large keratocytes with less contrast between the cell and clear stroma as compared to the adjacent keratocytes at day 0. The acellular zone persisted at day 9, but the number of reflective particles was reduced. Cells adjacent to the interface appeared similar to those at day 4 (Figure 4).

### Myofibroblasts are Absent and Fibronectin is Present at the Interface

**Figure 5:**
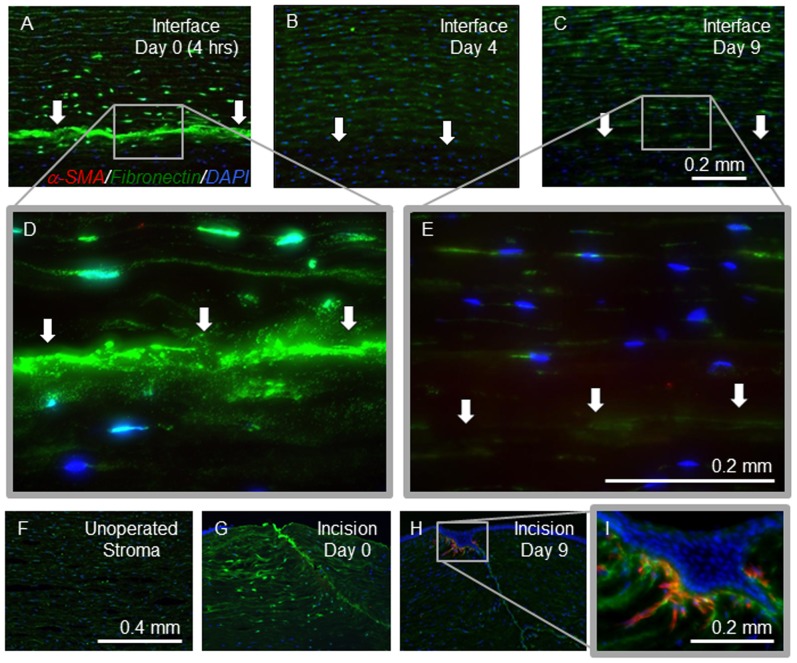
Immunohistochemistry for detection of alpha-smooth muscle actin (α-SMA) and fibronectin post-Descemet’s Stripping Automated Endothelial Keratoplasty (DSAEK). Corneal sections were stained with antibodies against α-SMA (red) to label myofibroblasts, antibodies against fibronectin (green), and 4′6′-diamidino-2-phenylondole dihydrochloride (DAPI) (blue) was used to label cell nuclei. (**A–C**) Photomicrographs of *ex vivo* cat corneal sections of the graft-host interface (arrows) on post-operative days 0 (**A**), 4 (**B**), and 9 (**C**)(scale bar for A – C = 0.2 mm). (**D and E**) The graft-host interface on days 0 (**D**) and 9 (**E**) (scale bar for D & E = 0.2 mm). (**F**) The central stroma of an unoperated cat cornea demonstrated an absence of α-SMA and mild diffuse fibronectin staining. (**G and H**) Incisional paracenteses wounds on day 0 (**G**) and day 9 (**H**) (scale bar for F – H = 0.4 mm). (**I**) An incisional paracentesis wound on day 9 at high magnification (scale bar for I = 0.2 mm). Note the lack of α-SMA staining at the graft-host interface (**C and E**), but positive α-SMA staining at the incisional wound (**H and I**) on day 9. On day 0, 4 hours after DSAEK, fibronectin staining was present extracellularly along the interface and also appeared to co-localize with DAPI in the cells of the adjacent host stroma (**A and D**). On day 9 post-DSAEK, there was faint fibronectin staining near the host stromal cells, but the interface fibronectin staining is much fainter and more consistent with the unoperated control.

Low magnification images of the DSAEK graft-host interface of corneal sections stained with antibodies against α-SMA, fibronectin, and DAPI are demonstrated at day 0 (Figure 5A), day 4 (Figure 5B), and day 9 (Figure 5C). In addition, higher-magnification images of the interface at day 0 (Figure 5D) and day 9 (Figure 5E) are also demonstrated. No α-SMA positive staining was observed at the graft-host interface at any of the early time-points examined (Figure 5A–E). However, positive staining was observed in peripheral small arterioles in all eyes at all time-points confirming successful staining in each section. Incisional paracenteses wounds made at the time of surgery served as positive surgical controls and revealed increased cellularity and positive α-SMA staining on day 9 (Figure 5H and I). Immunohistochemical staining of fibronectin revealed a layer of fibronectin along the entirety of the interface at day 0, 4 hours after the end of the DSAEK surgery (Figure 5A and D). This staining was much more intense and localized than the fibronectin staining observed in normal unoperated cat corneas (Figure 5F), and more consistent with the staining observed along the incisional wound at day 0 (Figure 5G). Fibronectin also stained positively within or around DAPI positive cells immediately adjacent to the interface and extending into the host stroma toward the epithelium (Figure 5A and D). The fibronectin staining associated with the neighboring cells subsequently diminished and at days 2, 4, 6, and 9, (Figure 5B, C, and E) was consistent with that observed in unoperated cat corneas (Figure 5F). Similarly, the interface fibronectin staining present at 4 hours disappeared and was not seen at subsequent time points (Figure 5B, C, and E).

### TUNEL Positive Cells are seen at the Interface Immediately Post-DSAEK

**Figure 6:**
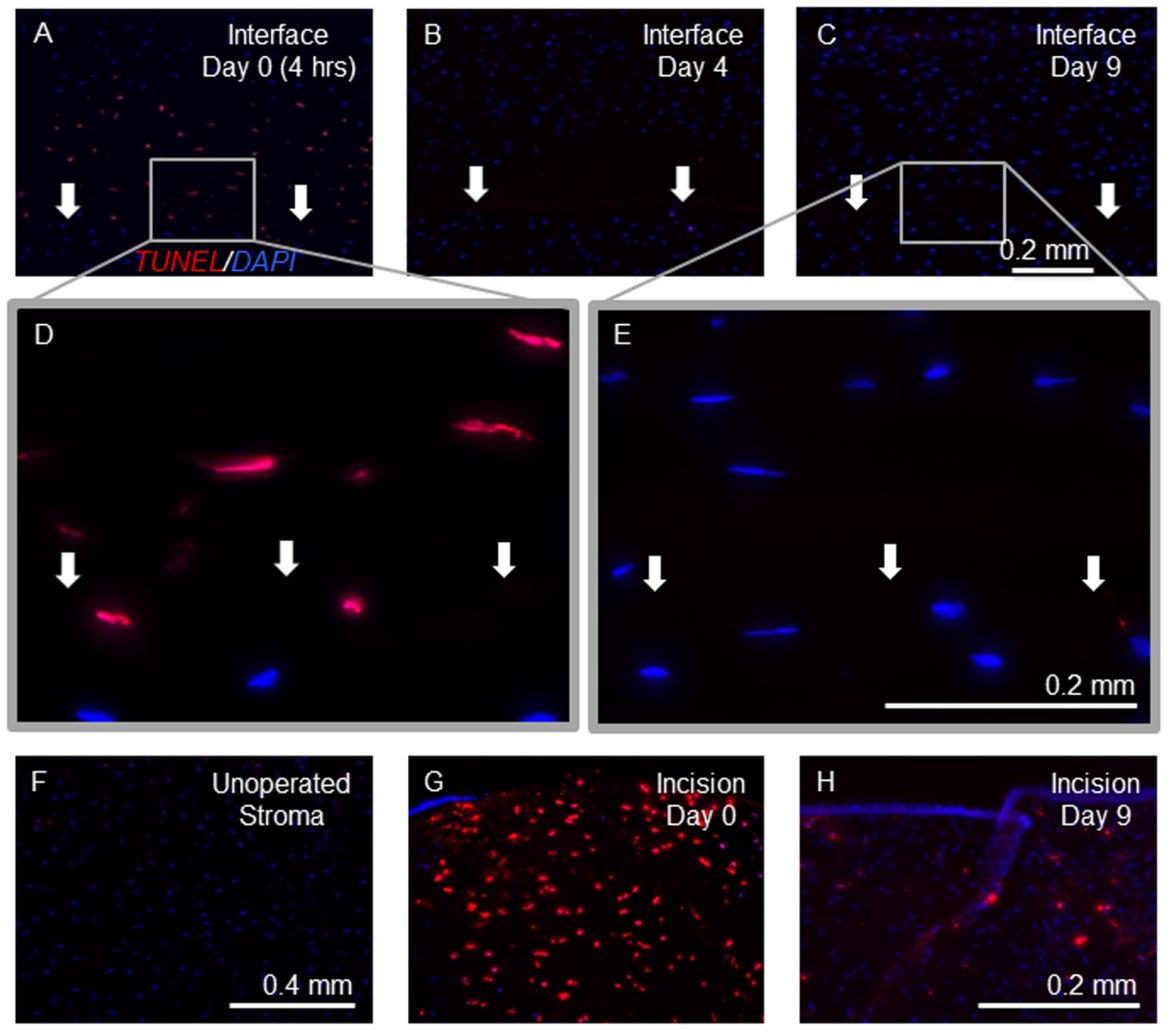
Immunohistochemistry for detection of Terminal deoxynucleotidyl transferase dUTP nick end labeling (TUNEL) post-Descemet’s Stripping Automated Endothelial Keratoplasty (DSAEK). Corneas were labeled using a TUNEL assay for apoptotic cells (red), and with DAPI (blue) to identify cell nuclei. (**A–C**) Photomicrographs of *ex vivo* cat corneal sections of the graft-host interface (arrows) on post-operative days 0 (**A**), 4 (**B**), and 9 (**C**) at (scale bar for A–C = 0.2 mm). (**D and E**) The graft-host interface on days 0 (**D**) and 9 (**E**) at higher magnification (scale bar for D and E = 0.2 mm). (**F**) An unoperated cat cornea at 10× shows an absences of TUNEL staining (scale bar for F and G = 0.4 mm). (**G and H**) Incisional paracenteses wounds at low magnification on day 0 demonstrated increased cellularity and intense TUNEL staining of most cell nuclei (**G**) and day 9 showed similar cell density with a smaller percentage of cell nuclei with positive TUNEL staining (**H**) (scale bar for H = 0.2 mm). Note the positive TUNEL staining at the graft-host interface and extending into the host stroma at day 0 (**A and D**) and its absence at the interface at subsequent time-points (**B, C, and E**). At time-points beyond day 0, there is an area of absent DAPI staining in a similar distribution to that of the positive TUNEL staining on day 0 (**B, C, and E**).

Low magnification images of the graft-host interface of corneal sections stained with TUNEL assay and counterstained with DAPI are demonstrated at day 0 (Figure 6A), day 4 (Figure 6B), and day 9 (Figure 6C). Additional higher-magnification images are also provided at day 0 (Figure 6D) and at day 9 (Figure 6E). TUNEL staining was positive on day 0, 4 hours post-DSAEK (Figure 6A and D), in a similar distribution to the fibronectin staining (Figure 5A and D). Specifically, cells immediately adjacent to the interface and extending into the adjacent host stroma stained positively for both fibronectin and TUNEL at the 0 day time point. Again similar to the fibronectin staining obtained and shown in Figure 5, TUNEL staining decreased subsequently and remained negative in the interface zone and adjacent tissues at the subsequent time-points (days 2, 4, 6, and 9) (Figure 6 B, C, and E), returning to the appearance of unoperated cat corneas which demonstrated no TUNEL staining (Figure 6F). Incisional corneal wounds show an abundance of TUNEL positive cells near the incision at day 0 (Figure 6G) with decreasing intensity and density of TUNEL positive cells 9 days later (Figure 6H). DAPI counterstain on days 2, 4, 6, and 9 revealed an acellular zone in the same distribution as the previously positive intracellular TUNEL and fibronectin staining, which decreased in thickness with time (Figure 6B, C, and E). On day 2, the acellular zone occupied the entire length of the graft-host interface and varied in thickness between 5 and 30% of the host stromal thickness. Between days 2 and 9, the acellular zone appeared to decrease in size, so that by day 9, although it was still visible, the acellular zone was much less pronounced, with some areas of the interface exhibiting relatively normal cell densities. Throughout the time points, there was some regional variability in the thickness of the acellular zone. It is notable that the incisional wound demonstrated the greatest number and largest area of TUNEL-positive cells 4 hours after the procedure (Figure 6G). By 9 days later, the number and intensity of TUNEL-positive cells had decreased significantly, although some staining persisted in cells adjacent to the incisional wound (Figure 6H).

## Discussion

### The Cat Model of DSAEK: an Opportunity to Study The Early Corneal Wound Healing Response

While *in vivo* optical analyses post-DSAEK have been performed [5]–[7], human tissue appropriate for a histologic correlation of the early post-operative biological alterations that may be causing corneal haze is difficult to obtain. Most human specimens of endothelial keratoplasty are available as a result of graft failure [20]–[23], which makes them inherently flawed in attempting to study normal post-operative changes.

In the present study, we used a feline model of DSAEK with allograft donor tissue to overcome these challenges. This model allowed us to correlate *in vivo* imaging findings with histological analyses at early time-points following a standardized DSAEK procedure with regimented perioperative treatment and follow-up. The cat was selected as the model for this study, in part because its corneal curvature, size, thickness, and histological structure are more similar to humans than those of most other mammals. [24] Rabbits are also frequently used for corneal research, but the regenerative capacity of the rabbit endothelium makes it less ideal for studying DSAEK. [25] The cat is a fairly good model for DSAEK because its corneal shape and anterior chamber depth make the surgery technically possible in a manner that approximates the procedure normally performed in humans, and because the feline endothelium, like the human endothelium, has a limited regenerative capacity. [26], [27] In addition, cats have been used previously to study corneal disease including *in vivo* models of Fuchs’ endothelial dystrophy (Brunette I. IOVS 2012; ARVO E-Abstract 330 and Haydari MN, et al. IOVS 2012; ARVO E-Abstract 3640). [28], [29] The cat model has also been used to further our understanding of ophthalmic surgical interventions, including keratoplasty.[30]–[34] Histological correlates have demonstrated keratocyte activation and apoptosis adjacent to wounds following penetrating keratoplasty. [33] Cats have also played a role in the development of endothelial keratoplasty, with seminal work on early endothelial keratoplasty techniques by Melles in 1998 including a cat model. [35] Finally, cat models have been extensively utilized to characterize corneal wound healing and its impact on ocular optics[36]–[38] as well as the impact of pharmacological agents used to modify these responses following laser refractive surgery. [39].

The major disadvantage of the cat model of keratoplasty was the exaggerated delayed immune response we observed. Species variability in immune response to intraocular insult or exposure has been previously documented. [40] The cat eye is particularly reactive with one study indicating that intracameral air injection alone was sufficient to cause an intraocular reaction in cats. [41] In our study, the immune response limited our ability to obtain useful data beyond the 9 days post-operative time-point. Our pilot experiments showed this response to be consistently apparent by 14–15 days post-operatively, and sometimes starting as early as 9 days post-DSAEK. In all cases, it was characterized by cells and fibrin-like material in the anterior chamber, as well as keratoprecipitates and hypercellular retrocorneal membranes. Ultimately, the membranes would result in scrolling and detachment of the graft and subsequent host corneal edema. This reaction following penetrating keratoplasty utilizing allograft tissue in felines has been documented by others. [31], [42].

Despite this limitation, we were able to successfully perform DSAEK in the cat and achieve clear grafts that lasted through 9 days post-operatively. We believe that if early myofibroblast differentiation were occurring at the interface, it would have been identified by our study. Eyes that were followed for up to 22 and 27 days in our pilot studies did not demonstrate α-SMA positivity at the interface (data not shown because of potential confounding of immune response; whereas, α-SMA-staining was clearly noted at the incisional site by 9 days. In incisional studies of rabbit corneas, α-SMA-staining of cells localized to the incision is clearly identified by day 3 with intense staining by day 7. [15] We anticipate that the time course for myofibroblast transformation in DSAEK to be more similar to that occurring with incisional wounds than that of photorefractive keratectomy based on the mechanism of injury, but also note that cats have a faster fibrotic response than that noted in rabbits. [39], [43] Thus we feel we were able to adequately assess the acute postoperative changes in corneal haze as well as the biological changes that might be driving them in a set of 10 successful DSAEK grafts.

### The DSAEK Interface Exhibits more Backscatter Reflectivity than Surrounding Tissues

We used OCT to quantify post-operative clinical corneal haze, which is defined as an observable opacification of the cornea. OCT generates images using low-coherence interferometry to compare the delay of tissue reflectance within the tissue being imaged against a reference reflection. [44] While forward light scatter is what ultimately impacts visual performance, and its functional correlate, referred to as straylight, has been studied in human subjects who are post-DSAEK [45], [46], this test requires interpretation by study subjects, which would be difficult to do in cats. An alternative to measuring forward scatter is to quantify backscattered light from high resolution, broad, cross-sectional, tomographic images of the cornea *in vivo*. Such OCT images were used as an indirect measure of corneal haze, assuming that changes in the cornea which induce backscattered light also likely scatter light in the forward direction. [47] Our OCT data showed significant increases in backscattered light at the DSAEK graft-host interface at Day 0 (4 hours post-DSAEK), persisting out to 2/3, 4/5, and 6/7 days. These results corresponded with the presence of interface haze observed clinically by slit lamp in our cats at these early post-DSAEK time-points. The difference between the mean interface backscatter compared to adjacent mean host stroma became more significant over time through 6/7 days. During this same post-operative time period, the host stroma, donor stroma, and total corneal thicknesses were trending downwards, although these differences were not statistically significant. Nonetheless, it seems unlikely that corneal edema alone accounts for the observed increase in corneal reflectivity with time after surgery. Previous studies have similarly failed to correlate total corneal thickness post-DSAEK with light scatter or visual acuity. [45], [48] However, the literature provides conflicting results with respect to role of graft thickness in visual outcomes post-DSAEK.[45], [46], [48]–[52].

In a short-term study through 3 months post-DSAEK of human subjects, Uchino et al quantified corneal haze using a Scheimpflug camera and found a decrease in haze between 2 weeks and 3 months post-operatively. [53] While it has been demonstrated that visual acuity in humans continues to improve through 3 years post-DSAEK [3], the explanation for these continued improvements in visual acuity is not fully understood. However, prior work in our laboratory found that decreased light scatter, especially at the graft/host interface, played a prominent role in improving visual acuity during the first year post-DSAEK. [54] Therefore, minimizing the interface haze early on, may benefit both short and long-term visual outcomes.

### Myofibroblasts do not Appear to Account for Interface Haze Following DSAEK

In a study that used *in vivo* confocal microscopy to assess interface haze prospectively in patients who had DSAEK surgery, interface haze was greatest at 1 month and subsequently declined significantly over the follow-up, although some haze persisted at 6 months. [7] Our study evaluated the early changes post-DSAEK from 4 hours to 9 days post-operatively in a cat model and identified increased interface haze relative to the stroma over the duration of this time period. The sources of light scatter (e.g. fluid clefts, refractive index change, irregularities of the interface, cellular opacity) cannot be determined, nor can we understand their changing contributions with time. However, based on our study, it is apparent the myofibroblast transformation is not associated with the observed scatter during these early post-DSAEK time-points. In other injury models, such as incisional wounds and photorefractive keratectomy, myofibroblast differentiation was observed by 7 days [15], [55] and therefore we anticipated that if myofibroblast differentiation were occurring, positive staining would be observed during the time period studied. The wound healing response in other studied corneal injuries results in a densely hypercellular region in which keratocytes proliferate and differentiate into less transparent fibroblasts and myofibroblasts. [56], [57] In the present study, we were not able to assess the role of α-SMA-negative activated fibroblasts, which if present, may also contribute to increased scatter at the graft-host interface.

In our study, the incisional wounds we used as positive controls showed both increased cellularity and α-SMA staining at the 9 day post-operative time-point; however, no α-SMA was observed at the graft-host interface post-DSAEK in our cat eyes, despite increased interface haze. These histological findings are consistent with our results from *in vivo* confocal imaging, which showed an absence of cells at the interface and with human studies of DSAEK using *in vivo* confocal imaging at later post-operative time-points, in which no myofibroblasts were identified at the graft-host interface. [7].

Studies of post-LASIK flaps may provide insight into the lack of myofibroblastic response observed here. In LASIK, myofibroblasts are involved in corneal wound healing at the periphery of the flap where Bowman’s membrane has been disrupted, but are rarely identified in the center of the flap where Bowman’s membrane remains intact. [58] A rabbit study evaluated histological components of the LASIK flap and identified fibrotic wound healing along the flap margin marked by α-SMA positivity. Meanwhile keratocytes toward the center of the flap remained quiescent with minimal fibrosis at the interface. [59] Wilson et al. tested the hypothesis that breaks in Bowman’s membrane, which allow epithelial-stromal interactions to occur, are necessary to stimulate keratocyte proliferation and myofibroblasts differentiation. To test this hypothesis, they intentionally placed epithelial tissue in experimental LASIK flaps and found that control flaps, unlike the flaps with the imbedded epithelial tissue, had less apoptosis and cellular proliferation and lacked α-SMA staining. [60] The central aspect of the LASIK flap may be far enough away from the disruption in Bowman’s membrane to be partially protected from the milieu of cytokines, chemokines and growth factors released by the epithelium and present in the tear film. Similarly, the post-DSAEK interface may lack a typical fibrotic response because of the absence of adjacent breaks in the Bowman’s membrane, and because it is far removed from any such breaks (which were present at the incision site). Another alternative is that cellular proliferation and myofibroblast differentiation at the interface are both delayed beyond the timeframe evaluated in the present study.

A big difference between the DSAEK graft-host interface and the LASIK flap interface is that we and others observed haze at the DSAEK interface, while the LASIK flap appears to lack it. [61] A possible explanation for this may lie in the inherent differences between the LASIK and DSAEK wounds. While neither the laser nor microkeratome cuts respect a single lamellar plane, the LASIK flap re-apposes tissues from the same person and likely does so in the appropriate lamellar orientation. DSAEK opposes tissues from two different individuals without attention to the donor tissue’s previous rotational orientation. Additional tissue engineering research may be needed to ascertain the relative importance of lamellar orientation for preserving corneal clarity *in vivo*.

### Cell Death and an Acellular Zone Characterize the Early Post-DSAEK Interface

Instead of cellular proliferation and myofibroblast differentiation occurring at the interface, we observed substantial cell death and keratocyte depletion, with an acellular zone developing over the early post-DSAEK period. Interestingly, apoptosis has also been seen at the surgical interface created by the LASIK flap. [62] Keratocyte apoptosis is cited as a first step in corneal wound healing reactions [62], and it has also been linked to the modulation and termination of corneal wound healing reactions. [63] We did not investigate the cause of the observed apoptosis, but possible triggering events could include mechanical effects of stripping the Descemet’s membrane and posterior stromal irritation, direct exposure of the posterior stroma to the cytokine milieu of the aqueous humor, pressure effects induced by the air bubble, and immune mediators induced by the surgical procedure.

It is possible that the process of cell death and the presence of an acellular zone could contribute to changes in corneal clarity. Modern models of corneal transparency are based on the lattice theory proposed by Maurice in 1957. [64] While many variations in the theory exist today, most of them hinge on a similar premise; each collagen fibril scatters a small amount of light, but the arrangement of the fibrils leads to destructive interference of the scattered light, leaving only the light traveling in the forward direction. [65] Keratocytes are responsible for the production and maintenance of this complex collagen structure and extracellular matrix composition. Without these cells, the extracellular matrix and the nearby corneal structure may be altered. Loss of corneal keratocytes may make the lamellae prone to irregular distribution, edema, or fluid lakes and disruption of fibril orientation. Additionally, whether cells repopulate the tissue, their phenotype, and their impact on corneal clarity all remain important aspects of the healing process to characterize. Future efforts aimed at understanding keratocyte death and repopulation near the interface may have important implications for visual recovery post-DSAEK.

### Extracellular Matrix Changes Occur with DSAEK

Fibronectin was identified at the interface and intracellularly at day zero (4 hrs. post-DSAEK), but did not persist at the subsequent post-operative time-points examined. Fibronectin is a glycoprotein that is thought to contribute to the wound healing process by providing scaffolding for cell migration or new extracellular matrix deposition, chemoattraction of immune cells, and opsinization of debris. [66] However, it has also been shown to influence cellular growth, differentiation and apoptosis through gene regulatory effects. [67] There are two main subsets of fibronectin; soluble plasma fibronectin (pFN) and insoluble cellular fibronectin (cFN). pFN is made in the liver and present in the circulating blood supply. It may also be present in the tear film or released from limbal blood vessels after corneal wounding. cFN is synthesized locally by fibroblasts and other cells that assemble extracellular matrix. [66] Only small amounts of fibronectin are present in normal, unwounded corneas. However, a previous study of corneal debridement, incision, and superficial keratectomy wounds *in vivo* suggest that both pFN and cFN are present to a much higher degree in wounded cornea. pFN appears first and is responsible for a layer of fibronectin that appears on the surface of wounds within 1 hour after wounding. Stromal cells then appear to be stimulated to synthesize greater amounts of cFN. [66] The source of the fibronectin that we observed along the interface on Day 0 is unclear. However, the interface fibronectin and the intracellular fibronectin that were generated, may be involved in extracellular matrix changes, wound remodeling, and collagen reorganization at the interface that could result in the decrease in interface corneal light scatter in longer-term studies of DSAEK. [5], [7], [8].

### Methodological Considerations

Our study was limited by the small number of animals and the short follow up post-DSAEK. The short post-operative follow up was due to the heightened immune response in our cat model, which led to early graft rejection and failure. However, based on studies of incisional wounds and PRK, the 9 day follow up time-point should have been sufficient to determine whether a fibrotic response was occurring at the interface. Another weakness of the present study was the small number of cats. While more of the quantitative trends we observed may have reached significance with the sacrifice of more cats, the major optical and histological outcomes of the study were so consistent, we did not feel sufficient additional insights would be gained to warrant sacrifice of more animals. Despite these shortcomings, it appears that myofibroblasts likely did not play an important role in the early interface wound healing process post-DSAEK in cats. Additionally, we identified keratocyte cell death and depletion and extracellular matrix changes, including the production and deposition of fibronectin, as important biological processes occurring at or near the interface.

## Conclusion

The apposition of the donor and host corneal stromata with DSAEK creates a tissue interface that induces light scatter. This interface has been noted at early post-operative time points in humans and has been found to improve with time post-DSAEK. Higher amounts of scatter have been correlated with poorer visual acuity and performance. Improvements in corneal light scatter from the interface have also been correlated with improvements in visual acuity post-DSAEK. In our cat model of DSAEK, interface haze was also prominent at early time periods post-DSAEK. However, it did not seem to be associated with myofibroblast differentiation. Instead, diffuse, increased fibronectin staining and death of adjacent stromal keratocytes were noted. Cellular death resulted in the subsequent development of an acellular zone in the host stroma, which showed evidence of cellular repopulation within the first 9 days post-DSAEK. A better understanding of the ways in which surgical manipulations during endothelial keratoplasty impact corneal biology may provide important insights for improving corneal clarity and especially interface haze post-DSAEK.
